# Supplementary material for: Social Motor Priming: when offline interference facilitates motor execution
Source: PeerJ. 2019 Oct 7;7:e7796. doi: 10.7717/peerj.7796 (PMC6786249; doi:10.7717/peerj.7796)
Supplement: Table S1 — Reaction Time (RT) data show the temporal delay between the ‘Go’ signal and movement onset (i.e., the time at which the tangential velocity of the wrist marker crossed a threshold of 5 mm/s and remained above it for longer than 500 ms). Movement Time (MT) data show the time interval between reaching onset and end of grasping (i.e., the time at which the hand opening velocity crossed a threshold of 5 mm/s after reaching its minimum value and remained above it for longer than 500 ms). Maximum Grip Aperture (MGA) data show the maximum distance reached by the 3D coordinates of the thumb and index finger for each individual movement. [file peerj-07-7796-s001.docx]

|  | RT | | | | | | MT | | | | | | MGA | | | | | |
| --- | --- | --- | --- | --- | --- | --- | --- | --- | --- | --- | --- | --- | --- | --- | --- | --- | --- | --- |
|  | INTERACTIVE | | NON-INTERACTIVE | | BASELINE | | INTERACTIVE | | NON-INTERACTIVE | | BASELINE | | INTERACTIVE | | NON-INTERACTIVE | | BASELINE | |
| Participants | PG | WHG | PG | WHG | PG | WHG | PG | WHG | PG | WHG | PG | WHG | PG | WHG | PG | WHG | PG | WHG |
| 1 | 251,50 | 214,36 | 135,20 | 392,33 | 200,09 | 250,24 | 1901,90 | 1060,71 | 1262,36 | 1472,86 | 1323,38 | 1157,14 | 60,24 | 130,37 | 98,65 | 132,82 | 55,84 | 125,41 |
| 2 | 238,11 | 145,45 | 178,87 | 304,87 | 183,53 | 180,65 | 2020,46 | 1267,37 | 1513,90 | 1640,95 | 1850,48 | 1202,60 | 131,25 | 107,31 | 101,43 | 104,84 | 117,07 | 137,09 |
| 3 | 287,35 | 143,53 | 238,11 | 373,53 | 278,87 | 187,65 | 2047,10 | 1420,66 | 1427,03 | 1617,62 | 1743,33 | 1750,48 | 132,74 | 146,40 | 90,95 | 141,51 | 137,09 | 138,02 |
| 4 | 285,11 | 161,07 | 290,27 | 358,00 | 230,80 | 199,11 | 1480,12 | 1428,96 | 1497,10 | 1464,76 | 1423,81 | 1640,95 | 130,06 | 140,72 | 74,70 | 148,79 | 124,11 | 143,64 |
| 5 | 399,11 | 213,73 | 282,30 | 360,62 | 304,87 | 290,27 | 2472,86 | 1397,10 | 1947,10 | 2428,96 | 1901,90 | 1378,10 | 140,72 | 121,00 | 107,39 | 124,68 | 129,83 | 129,02 |
| 6 | 295,81 | 264,87 | 286,53 | 353,15 | 277,13 | 345,70 | 1621,62 | 1820,46 | 1325,87 | 1717,62 | 1666,67 | 1901,90 | 125,94 | 130,72 | 113,96 | 132,43 | 129,02 | 132,82 |
| 7 | 371,41 | 221,20 | 253,33 | 341,11 | 235,20 | 347,24 | 2047,10 | 1382,05 | 1370,85 | 1482,38 | 1526,67 | 1425,71 | 118,98 | 114,42 | 113,64 | 121,51 | 88,49 | 104,84 |
| 8 | 360,62 | 261,73 | 279,13 | 314,81 | 286,53 | 319,22 | 2228,57 | 1383,01 | 1498,46 | 1750,48 | 1619,52 | 1517,62 | 116,70 | 128,79 | 89,02 | 135,63 | 55,30 | 141,51 |
| 9 | 398,81 | 266,53 | 253,15 | 378,87 | 392,33 | 282,30 | 1302,60 | 1280,12 | 1328,76 | 1428,96 | 1374,29 | 1219,52 | 127,39 | 124,68 | 94,43 | 137,29 | 141,51 | 141,90 |
| 10 | 270,14 | 238,00 | 251,20 | 323,93 | 253,33 | 267,49 | 1364,76 | 1002,51 | 1238,42 | 1640,95 | 1221,43 | 1102,86 | 123,96 | 114,43 | 104,18 | 90,37 | 90,95 | 101,56 |
| 11 | 379,78 | 277,40 | 173,53 | 263,35 | 365,00 | 394,70 | 1436,25 | 1212,36 | 1421,49 | 2472,86 | 1472,86 | 1382,86 | 124,12 | 124,18 | 135,63 | 127,31 | 79,30 | 129,68 |
| 12 | 361,16 | 202,20 | 150,33 | 334,36 | 341,47 | 253,18 | 1303,81 | 1328,96 | 1091,43 | 1383,01 | 1328,57 | 1260,00 | 121,51 | 105,63 | 121,51 | 126,40 | 47,07 | 124,55 |
| 13 | 283,18 | 221,47 | 245,70 | 316,27 | 279,13 | 250,60 | 1247,51 | 1091,43 | 1292,50 | 2160,71 | 1214,29 | 1228,57 | 135,63 | 129,43 | 105,63 | 124,55 | 124,55 | 131,43 |
| 14 | 353,15 | 254,73 | 180,65 | 379,13 | 316,27 | 270,50 | 1520,66 | 1378,93 | 1123,21 | 1423,81 | 1482,38 | 1658,10 | 137,29 | 126,45 | 107,29 | 120,24 | 120,24 | 127,73 |
| 15 | 281,03 | 320,33 | 199,11 | 353,33 | 299,40 | 274,83 | 1478,10 | 1147,50 | 1436,25 | 1437,37 | 1464,76 | 1303,81 | 119,77 | 101,43 | 128,02 | 90,54 | 40,54 | 98,65 |
| 16 | 288,48 | 276,00 | 173,53 | 350,33 | 323,93 | 275,60 | 1750,48 | 1374,11 | 1336,43 | 1747,50 | 1519,52 | 1290,95 | 110,40 | 114,59 | 103,64 | 112,41 | 69,59 | 112,41 |
